# The Rethinking Clinical Trials (REaCT) Program: A Pragmatic Research Strategy to Improve Cancer Care for Patients, Caregivers, and Healthcare Systems

**DOI:** 10.3390/curroncol32090484

**Published:** 2025-08-29

**Authors:** Marie-France Savard, Mark Clemons, Sharon F. McGee

**Affiliations:** 1 Department of Medicine, Division of Medical Oncology, The Ottawa Hospital and the University of Ottawa, Ottawa, ON K1H 8L6, Canada; mclemons@toh.ca (M.C.); shmcgee@toh.ca (S.F.M.); 2 Cancer Therapeutics Program, Ottawa Hospital Research Institute, Ottawa, ON K1H 8L6, Canada

**Keywords:** pragmatic trials, quality of life, patient-centered care

## Abstract

Cancer care has become increasingly complex, expensive, and inaccessible, with patients often exposed to increased treatment-related harms for marginal benefits. Pragmatic clinical trials offer a solution by conducting real-world studies that evaluate dose optimization, toxicity, quality of life, and resource utilization. Pragmatic trials can also address the efficacy–effectiveness gap: the poorer outcomes and greater toxicity observed in everyday practice compared to those reported in many clinical trials. The Rethinking Clinical Trials (REaCT) program was designed to conduct patient-centered practice-changing research by involving patients, their families, and healthcare providers in the design of inclusive, real-world clinical trials. The REaCT process starts with surveys and systematic reviews to identify knowledge gaps and uses this information to design pragmatic clinical trials that address these deficits. Since 2014, the program has conducted 17 patient and 17 healthcare provider surveys with 2298 and 1033 responses, respectively. With these results, the program has performed 22 systematic reviews. These surveys and systematic reviews have resulted in 19 completed and 8 ongoing REaCT clinical trials that have recruited over 5000 patients from across Canada. Here, we present some of the practice-changing research conducted by the REaCT program and address challenges facing the growth of pragmatic research.

## Abstract (plain language summary)

Cancer care today is often complicated, costly, and difficult for patients to access. Many treatments can cause serious side effects, yet only offer small benefits. The REthinking Clinical Trials (REaCT) program aims to improve this by running practical, real-world studies that help doctors and patients make better treatment choices. These studies focus on everyday issues like finding the right dose, reducing side effects, improving quality of life, and using healthcare resources more wisely. Since 2014, REaCT has gathered insights from over 3000 patients and healthcare professionals and used this information to launch 27 clinical trials—19 completed and 8 still underway. These studies have involved more than 5000 patients across Canada. This article highlights some of REaCT’s most impactful research and the challenges of expanding this kind of patient-centered pragmatic approach.

## 1. Introduction

While major advances in cancer treatment have occurred because of clinical trials, this progress, when viewed globally, has rarely been patient-centered or equitable [1,2]. The reasons are multifaceted, but one key factor is the current research paradigm, which is largely sponsored by pharmaceutical companies and focuses on evaluating drug efficacy in highly selected patient populations that rarely reflect those seen in real-world clinical practice. The efficacy–effectiveness gap describes the differences in outcomes seen in clinical trials compared with routine clinical practice [3]. Quite simply, survival is usually shorter and toxicity greater in everyday practice than in clinical trials [4]. Lack of recognition of the efficacy–effectiveness gap may cause patients, clinicians, and policymakers to have unrealistic expectations of the benefits and harms of therapy across a range of clinical scenarios [5]. Furthermore, rising healthcare costs, due mainly to new expensive treatments, are creating an unsustainable situation that has resulted in substantial disparities in cancer care worldwide [6].

Despite the shift from classical chemotherapy to molecularly targeted agents, the paradigm for anticancer drug development has remained largely unchanged. Phase 3 clinical trials, typically those conducted for regulatory approval, continue to rely on doses established in early-phase studies based on the maximum tolerated dose. These doses are often much higher than required for maximal target inhibition and maximum benefit [1,2], resulting in avoidable side effects, increased costs, and reduced access to potentially effective treatments [7]. Therefore, substantial savings could be achieved by optimizing current registered interventions [8,9]. Organizations such as The Optimal Cancer Care Alliance (OCCA), Choosing Wisely, and others promote optimal drug dosing and the avoidance of unnecessary procedures and treatments. Regulatory agencies such as the U.S Food and Drug Administration (FDA) [10] and European Medicines Agency (EMA) [11] have recently recognized this problem, and the FDA has indicated that it will require dose optimization as new agents are developed [12]. However, the FDA cannot require this to be undertaken for drugs already approved, and there is no incentive for pharmaceutical companies to do so. Thus, optimization of dose, schedule, and treatment preferences for approved treatments requires publicly supported research strategies and funding. While pragmatic trials are not a new concept, they could play a critical role in the generation of real-world evidence on the true benefits and risks of approved treatments [13,14].

The Rethinking Clinical Trials (REaCT) program was developed to address these issues by promoting patient-centered, evidence-based, and high-quality cancer care. To support this goal, REaCT has undertaken a series of patient and healthcare-provider surveys and systematic reviews. This information has been used to design pragmatic clinical trials for cancer patients, with over 5000 patients enrolled at centers across Canada. REaCT trials compare different approved treatments and/or evaluate optimal dosing of registered anti-cancer treatments. The program aims to inform patients, healthcare providers, and healthcare systems about treatment options that offer the best clinical outcomes while minimizing toxicity and cost. The REaCT program’s foundation, successes, and challenges are presented below.

## 2. The Rethinking Clinical Trials (REaCT) Program

The REaCT program was created in 2014 based on observed needs and gaps in daily patient care. While clinical trials have led to significant advances in cancer management, patients, families, and healthcare providers are often left navigating multiple treatment options without clear guidance on which offers the best patient-centered outcomes (e.g., quality of life, disease-free or overall survival), treatment-specific side-effects, and associated costs. Furthermore, treatment decisions were often based on anecdote and provider preference, rather than evidence.

### 2.1. Stakeholder Surveys: Selection of Relevant Questions and Endpoints

**Figure 1:**
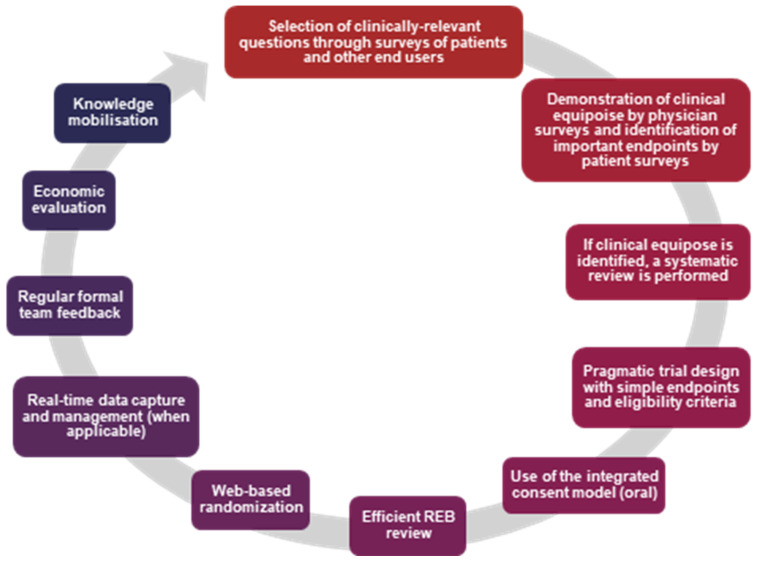
Step-by-step overview of the REaCT methodology.

Recognizing the challenges and limitations of clinical trial design and conduct, the REaCT program sought to develop an approach focused on patient-centered, evidence-based, and pragmatic research strategies [15]. The REaCT team is composed of experts in research methodology, biostatistics, health economics, and patient-reported outcomes. The processes involved in performing REaCT studies are shown in Figure 1. To ensure that a research program is relevant and patient-centered, the first step is to survey patients, their families, and healthcare providers [16]. These surveys help identify key clinical questions and reveal variations in practice patterns. The surveys also incorporate patients and physicians in clinical trial design by identifying study endpoints that are clinically meaningful to both and would be sufficient to drive a change in practice. To date, the REaCT program has performed 17 patient and 17 healthcare provider surveys, with a total of 2298 and 1033 responses, respectively.

### 2.2. Systematic Review: Identifying Knowledge Gap

If the survey identifies potential clinical questions of importance to patients and healthcare providers, as well as clinical equipoise (i.e., disagreement among the community of experts about the preferred management), then a systematic review, and if possible, meta-analysis, of the proposed research question is undertaken [17]. This comprehensive approach to reviewing the available evidence is well-defined [18] and allows investigators to assess the quality of data that have been generated, incorporate key findings into study design, and ensure that research addresses knowledge gaps. The systematic review also enables investigators to learn from the challenges faced by previous investigators and to incorporate these lessons into the design of future studies. The REaCT program has performed 22 systematic reviews evaluating standard of care interventions for the management of breast cancer (*n* = 15), all tumor sites (*n* = 4), breast and prostate cancers (*n* = 2), and prostate cancer (*n* = 1).

### 2.3. Pragmatic Design: Broad Eligibility Criteria and Oral Consent

Using the information from surveys and systematic reviews, the REaCT team meets to decide if there is a need to perform a clinical trial to answer a specific clinical question. If so, REaCT methodology is applied to design a pragmatic, real-world clinical trial to address the question and inform clinical practice. Critical to this is the identification and removal of the many barriers to clinical trial participation that patients and healthcare providers face [19,20]. These include concerns about randomization, mistrust of the research process, complex protocols, fear of side effects, and a lack of awareness about available trials, factors that must be addressed to ensure equitable and meaningful patient participation.

Broad eligibility criteria help make studies more inclusive and representative of real-world patient populations, thereby improving the generalizability of the results [21]. The traditional written consent process is replaced by an integrated consent model, which includes verbal consent and allows patients to be approached about trials during in-person or virtual visits with their treating oncologist [22]. The patient receives a consent template outlining the key details of the study. After their questions are addressed, the healthcare provider documents the discussion in the patient’s electronic medical record. A signed consent form is not required, and because the conversation mirrors a typical real-world consultation, it minimizes delays in the clinic workflow [15].

Once oral consent is obtained, randomization is web-based and can be performed immediately in the clinic by the oncologist to avoid unnecessary return visits. An electronic database captures data in real time while minimizing reporting tasks for patients and physicians by focusing only on outcomes that are most relevant to their needs. Follow-up and clinical investigations during the study are based on physician recommendations and the standard of care, with no additional trial-mandated visits or testing [15].

### 2.4. REaCT Program Metrics

**Figure 2:**
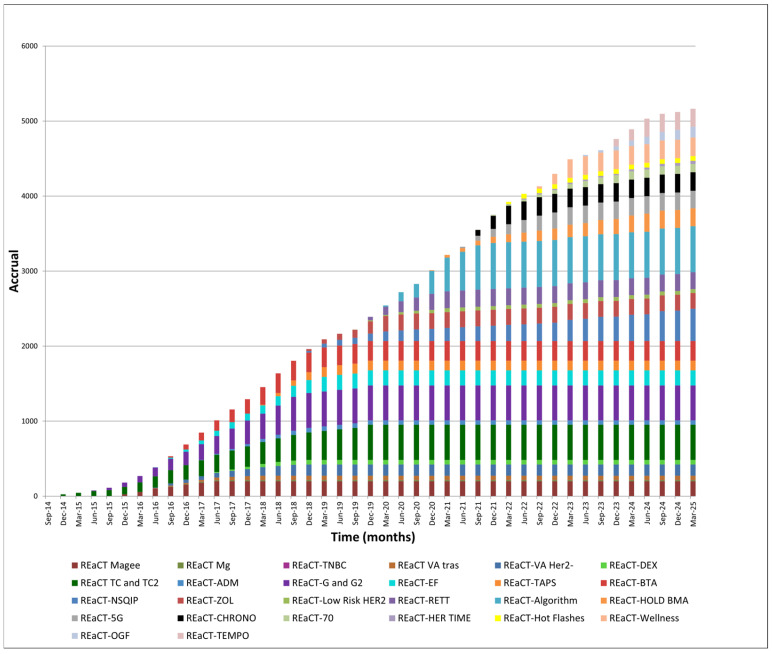
REaCT clinical trials’ accrual over time.

Since 2014, the REaCT program has completed 17 clinical trials and currently has 10 ongoing, enrolling over 5000 patients across 21 Canadian cancer centers (Figure 2). Trials have addressed clinical questions across surgery, radiotherapy, and medical oncology, as well as supportive care, cardiac imaging, central line use, and palliative care. Patients have participated in these studies with a range of cancers, including breast, gastrointestinal, genitourinary, and primary brain malignancies. Completed and published trials are shown in Table 1. Table 2 shows trials that are closed to accrual or ongoing.

## 3. REaCT Clinical Studies

The majority of completed REaCT trials have impacted local practice and have raised interest internationally. For example, in the adjuvant setting, it has been shown that women with early breast cancer can safely begin endocrine therapy alongside radiotherapy (REaCT-RETT) [41], and at whichever time of the day (morning or evening) they prefer (REaCT-CHRONO) [27]. Regarding the use of granulocyte-colony stimulating factors (G-CSF), 5 days of short-acting G-CSF is sufficient to prevent febrile neutropenia or treatment-related hospitalization (REaCT-G & G2) [32,33]. No difference in bone pain or quality of life was found when 5 days of short-acting G-CSF was compared to long-acting G-CSF (REaCT-5G) [29]. Cardiac monitoring can be de-intensified from 3-monthly to 4-monthly while on adjuvant trastuzumab (REaCT-EF) [39]. Patients forgetting to take previous doses of dexamethasone before docetaxel could safely receive a fixed 8 mg oral dose, thereby reducing delays in starting docetaxel infusion (REaCT-DEX) [37]. In the metastatic setting, we and others have shown that dosing of bone-targeted agents every 12 weeks was non-inferior to 4-weekly doses and is now our standard of care (REaCT-BTA) [43,44,46]. Overall, these trial results help optimize clinical practice by reducing costs, unnecessary testing, and follow-up visits. Their global impact, however, remains to be fully determined.

Not all trials have achieved their planned goals. For these studies, the major barrier has been poor physician engagement with the study question. For example, some REaCT trials had slow accrual that prevented the launch of larger, definitive pragmatic trials. Examples include REaCT-MG (evaluating two oral magnesium supplements) [42] as well as REaCT-VA HER2-negative [25] and REaCT-VA HER2-positive [26] (evaluating vascular access strategies for chemotherapy administration).

## 4. Challenges of (REaCT) Pragmatic Trials

### 4.1. Accessing Funding

While there is extensive support for pragmatic trials, challenges exist. As outlined in Table 1, several studies demonstrated the superiority of less intensive treatments in terms of toxicity, quality of life, and cost. It is not surprising that pharmaceutical companies, driven by monetary incentive, are not interested in funding this type of research. Paradoxically, while many trials that evaluate reduced dosing save more money than they cost to run, it is a challenge to persuade payers to finance them [14]. Our applications for larger peer-reviewed grants have faced the barrier that REaCT trials rarely incorporate new molecular markers and, therefore, are perceived as being “mundane” and not innovative. In addition, some reviewers have appreciated the potential impact of a particular study but indicated that the study should be funded by taxpayers. To date, most REaCT trials have been funded through patient donations, hospital foundations, and small research organizations.

### 4.2. Competition from Pharmaceutical Trials

Given that pragmatic clinical trials are conducted on a limited budget, there is low-level financial support for participating centers and patients. Industry-sponsored trials typically provide sizable funding to cover the cost of resources, and this allows centers to profit from pharmaceutical trial participation. There can also be competition between pharmaceutical and pragmatic trials for a specific patient population, and the former typically wins due to the financial incentive. While (REaCT) pragmatic trials do not prevent patients from participating in other research studies, pharmaceutical trials do.

### 4.3. Publishing Results

Publishing the results of pragmatic trials can also be challenging, as editors and reviewers may not fully understand or value their patient-centered endpoints and pragmatic design and conduct. These studies are often mischaracterized as exploratory trials aimed at demonstrating benefit under ideal conditions, which is far from their actual intent. Thus, reports of pragmatic trials are often published in journals with lower impact, reducing their exposure and, thus, adoption in practice [47].

### 4.4. Limited Uptake of Dose Optimization Studies in Clinical Practice

It is common in real-world practice for healthcare providers to reduce the doses of drugs and increase the intervals between treatments to ensure patient safety and tolerability, especially for older patients and those with comorbidities [48]. These “off-label” doses and intervals, even when supported by evidence that they are equally effective and less toxic, are not incorporated into product monographs, either because they were not supported by clinical trials or because there is no obligation for pharmaceutical companies to update monographs based on new studies. Also, the regulatory requirement to perform rigorous dose-optimization trials is often burdensome. For example, bisphosphonate therapies were initially approved for cancer patients with bone metastases every 4 weeks, resulting in significant toxicity, despite previous research indicating their long half-life and prolonged impact on bone turnover [49]. It was only much later that trials were performed showing that bisphosphonate treatment every 12 weeks was equally effective, but significantly less toxic [43,44,46].

Studies investigating reduced dose, schedule, or duration frequently face unique barriers that can impact patient and physician engagement and limit accrual. For patients and healthcare providers, there may be concerns about what may be perceived as “de-escalation” of treatment and an increased fear of disease progression/recurrence [50]. An example was the recent suspension of the BIG 19-02, Decrescendo trial [NCT04675827], which investigated a less intensive and toxic neoadjuvant systemic chemotherapy regimen for patients with early-stage HER2-positive breast cancer. The trial was suspended due to concerns that slow recruitment could undermine the scientific validity of the study and jeopardize its financial sustainability [51]. While modifying terminology from “treatment de-escalation” to “treatment optimization” is one step to combat this stigma, greater efforts are needed to persuade stakeholders that treatment optimization is important to maintain efficacy without added toxicity. Outside the context of a clinical trial, guidelines tend to recommend doses that are used in the pharmaceutical company-sponsored RCT that are registered with the FDA or EMA. Thus, healthcare providers may perceive an increased risk of litigation for prescribing therapies off-label.

Another barrier is the adoption of the results of treatment optimization studies into routine clinical practice. For example, the results of studies comparing 6 months of adjuvant trastuzumab with 12 months in early breast cancer show that shorter courses were associated with almost identical cancer outcomes with less toxicity [52,53]. The uptake of these findings has been limited, and they have not been incorporated into drug monographs or clinical practice guidelines.

## 5. Overcoming the Challenges of Pragmatic Trials

A key challenge for pragmatic trials evaluating fewer intensive therapies is the prevailing belief, often reinforced by pharmaceutical lobbying, that such studies must use a non-inferiority design [54]. However, non-inferiority designs have important limitations. They rely on an arbitrary non-inferiority margin, which can lead to different conclusions in the same population, depend on benefit estimates from trials conducted under ideal conditions, and focus on a single primary efficacy outcome that does not account for treatment-related harms [55]. Furthermore, using a classical non-inferiority design, the study sample size required to show “non-inferiority” is 4 to 5 times bigger than the sample size of the registration trial that uses a superiority design [55]. Larger sample sizes significantly impact the cost and duration of studies and can be impossible to achieve within the design and budget of a pragmatic trial.

Newer, more pragmatic designs are focusing on using outcomes to analyze patients rather than patients to analyze outcomes [56]. For instance, the desirability of outcome ranking (DOOR) and partial credit strategy have gained popularity in other fields of medicine, such as infectious disease [57]. Based on the concept of within-patient analyses and composite benefit:risk endpoints, the efficacy and safety outcomes are integrated first at the patient level; data are then summarized per intervention, and lastly, interventions are compared. Using these pragmatic designs, the required sample size is usually 50% smaller than the original classical non-inferiority design. Also, patients are fully engaged in the benefit:risk analyses by selecting the outcomes and assigning relative importance. These methods can therefore increase feasibility and better inform benefit:risk decision-making than conventional designs by reflecting the totality of the patient experience, benefits, and harms.

Another strategy is the near-equivalence approach that circumvents the need to perform a new randomized control trial by evaluating the acceptability of an alternative treatment relative to standard-of-care by using all the various types of available evidence, including efficacy, quality of life, toxicity, cost-effectiveness, pharmacokinetic, and pharmacodynamic data [54,58]. This utilitarian approach that goes beyond a simple cost-effectiveness analysis may improve accessibility to treatment strategies that have less financial and physical toxicity. Other potential means of reducing sample sizes include the use of artificial intelligence and the use of virtual controls [59].

### 5.1. Growing Organizational Support

Several organizations have been created to promote the importance of patient-centered, evidence-based care and research in oncology. The Optimal Cancer Care Alliance (OCCA) focuses on the reduced toxicity and substantial savings that can be achieved by optimizing the use of registered treatments and interventions [60]. An example of an evidence-based strategy to optimize drug dosing, supported by OCCA, was recognition that eating a meal prior to taking abiraterone for metastatic prostate cancer increases drug availability compared to taking it fasting, allowing patients to take lower doses, creating significant savings related to toxicity and cost [61,62]. Other examples of agents under investigation at a reduced dose intensity include nivolumab [59], pembrolizumab [63], bevacizumab [64], sotorasib [65], and ibrutinib [66].

The Common Sense Oncology (CSO) movement was launched in July 2023, with MASSIVE interest (>1 million page views on Twitter within 48 h of launch) (Booth, C., personal communication, 2023). This group has highlighted key challenges facing cancer care globally, with increasingly complex, expensive, and inaccessible treatment options, and treatment-related toxicities that often do not improve patient outcomes or quality of life. Common Sense Oncology is lobbying for cancer care to be more patient-centred and equitable and aims to achieve this through real-world evidence generation, evidence interpretation, and evidence communication [67].

The FDA Oncology Center of Excellence has recognized the importance of pragmatic research. In 2022, the FDA launched the FDA Project Pragmatic to integrate clinical trials with real-world clinical practice by promoting pragmatic trials [68]. Subsequently, Project 5 in 5 was initiated to identify five clinically relevant questions that can be answered over the next 5 years by performing pragmatic clinical trials to evaluate FDA-approved cancer treatments [69].

### 5.2. Novel Funding Models

Many organizations are leveraging public funding for treatment optimization studies based on the cost savings generated by less intensive dosing, frequency, and duration of treatment [14]. The Dutch phase 3 SONIA trial compared the use of CDK4/6 inhibitors as first versus second-line therapy for hormone receptor-positive metastatic breast cancer, demonstrating that second-line use of these expensive agents was associated with the same breast cancer-related outcomes of progression-free and overall survival [70]. Use of CDK4/6 inhibitors in the second rather than first line would equate to a yearly cost reduction of EUR 70 million for the Netherlands and a similar reduction in other countries [71]. Similarly, the Swiss Group for Clinical Cancer Research (SAKK) 96/12, REDUSE trial compared de-escalating from 4-weekly to 12-weekly denosumab in patients with bone metastases from breast or prostate cancers with similar outcomes [72]. In the UK, the REFINE trial is investigating giving single-agent immunotherapy less often to patients with advanced cancer to determine the impact on efficacy, toxicity, and quality of life [50]. Our REaCT Program will use patient donations to contribute to this latter initiative. It is our vision to foster international collaboration in the coming years to promote pragmatic trials.

## 6. Conclusions

The rapid pace of cancer drug discovery is to be commended; however, uncoordinated research efforts, which are predominantly pharmaceutical company-sponsored, have generated multiple standards of care that have not been compared, with limited quality of life data and few dose optimization studies. Pragmatic clinical trials provide a critical opportunity to address these issues to improve the quality and efficiency of care for the benefit of patients, caregivers, and the health system. Every trial conducted by the REaCT program has shown that performing fewer procedures and treatments was associated with equal effectiveness, less toxicity for patients, and considerable cost savings for the healthcare system. While the REaCT program has had many successes, challenges remain. We hope that increasing awareness of the importance of pragmatic research, combined with greater organizational support, funding, and engagement from clinicians and policymakers, will ensure that pragmatic clinical trials play a central role in shaping evidence-based, patient-centered cancer care.

## Figures and Tables

**Table 1:** REaCT clinical trials portfolio—completed and published trials.

Study Name Trial Number [Funding Source]	Number of Participants Enrolled (Number of Sites Opened)	Current Status	Key Findings
Adjuvant—surgical studies			
A randomized trial comparing alloderm-RTU with DermACELL in immediate subpectoral implant-based breast reconstruction (REaCT-ADM) [23] NCT03064893 [Internal funds]	62 (1)	Completed	No significant differences were observed in minor or major complications and drain duration between DermACELL over Alloderm-RTU.
Adjuvant—pathology studies			
A cohort study evaluating the impact of pre-emptive availability of PREDICT 2.1 results on ordering practices for Oncotype Dx RS (REaCT-Algorithm) [24] NCT04131933 [OICR Health Services Research grant]	602 (6)	Completed	Providing PREDICT 2.1 results and an educational intervention did not alter the ordering of an Oncotype DX. Routine ordering of molecular assays for patients with low clinical-risk disease is of poor value.
Adjuvant—vascular device studies			
A randomized pilot trial comparing vascular access strategies for early-stage breast cancer patients receiving non-trastuzumab containing chemotherapy (REaCT-VA HER2-negative) [25] NCT02688998 [Internal funds]	150 (2)	Completed	While meeting its a priori feasibility criteria for patient engagement, the slow accrual means that conducting a large pragmatic trial would require overcoming the barriers to physician recruitment.
A randomized trial comparing vascular strategies for patients receiving chemotherapy trastuzumab for early-stage breast cancer (REaCT-VA-HER2-positive) [26] NCT02632435 [Internal funds]	56 (1)	Completed	The study met its feasibility endpoints with respect to patient and physician engagement. However, the slow rate of accrual (56 patients in 2 years) means that conducting a large pragmatic trial would require additional strategies to make such a study possible.
Adjuvant—endocrine therapy and supportive care studies			
A pragmatic, randomized trial comparing morning versus evening dosing of endocrine therapy in patients with early-stage breast cancer (REaCT-CHRONO) [27] NCT04864405 [NOAMA Grant]	245 (2)	Completed	No significant difference in quality of life or adherence if endocrine therapy is taken in the morning or in the evening.
A prospective study investigating treatment-related vasomotor symptoms in patients with early-stage breast cancer (REaCT-Hot Flashes) [28] [AMS Healthcare Small Grant in Compassion and Artificial Intelligence, donations]	88 (2)	Completed	Baseline symptom severity and the directionality of change (improvement or deterioration of symptoms) influence the perception of clinically meaningful change among patients with breast cancer experiencing vasomotor symptoms.
Adjuvant—chemotherapy and supportive care studies			
A randomized trial comparing 5 days of filgrastim vs. pegfilgrastim for neutropenia prophylaxis in early breast cancer (REaCT-5G) [29] NCT04781959 [TOHAMO Innovation Grant]	233 (2)	Completed	No difference between 5 days of filgrastim and single dose of pegfilgrastim in terms of bone pain, health-related quality of life, chemotherapy delay, dose reduction, premature discontinuation, or chemotherapy-related deaths.
A study to determine the feasibility of using an integrated consent model to compare three standard of care regimens for the treatment of triple-negative breast cancer in the neoadjuvant/adjuvant setting (REaCT-TNBC) [30] NCT02688803 [Ottawa Hospital Department of Medicine Patient Quality and Safety Committee, SPOR Grant]	2 (1)	Completed	Feasibility was not met in this study, and it was closed.
A randomized study comparing tapering low dose dexamethasone to other standard of care therapies for taxane-associated pain syndrome (TAPS) in breast cancer patients (REaCT-TAPS) [31] NCT03348696 [Internal funds, Cancer Care Ontario Clinical Programs and Quality Initiatives Grant]	130 (2)	Completed	A tapering schedule of dexamethasone was associated with a brief reduction in docetaxel-associated symptoms, which was observed only during dexamethasone exposure and did not persist after discontinuation of the drug.
A randomized trial comparing schedules of filgrastim administration for primary prophylaxis of chemotherapy-induced febrile neutropenia in early-stage breast cancer (REaCT-G & G2) [32,33] NCT02428114 & NCT02816164 [CIHR-SPOR grant and a Cancer Care Ontario Clinical Programs and Quality Initiatives Grant]	466 (6)	Completed	Five days of filgrastim was non-inferior to 7/10 days in terms of febrile neutropenia or treatment-related hospitalization. Given the cost and toxicity of this agent, 5 days should be considered standard of care.
A randomized study comparing granulocyte-colony stimulating factors to antibiotics for primary prophylaxis of docetaxel–cyclophosphamide-induced febrile neutropenia (REaCT-TC & TC2) [34,35] NCT02173262 & NCT02816112 [Ottawa Hospital Department of Medicine Patient Quality and Safety Committee with matched funding from the Ottawa Hospital Division of Medical Oncology, SPOR Grant]	458 (4)	Completed	The primary endpoint of superiority of G-CSF over ciprofloxacin was not demonstrated. While there were reduced febrile neutropenia rates with G-CSF, there were no differences in chemotherapy dose delays/reductions or discontinuations. With the commonly used willingness-to-pay value of CAD 50,000/QALY, G-CSF use was not cost-effective compared to ciprofloxacin.
A randomized trial of individualized versus standard of care antiemetic therapy for breast cancer patients at high risk for chemotherapy-induced nausea and vomiting (ILIAD) [36] NCT02861859 [The Canadian Cancer Society Grant]	229 (3)	Completed	In patients at high personal risk of CINV, the addition of 5 mg daily of olanzapine to standard antiemetic therapy significantly improved the control of nausea and HR-QoL, with no unexpected toxicities.
A randomized trial comparing physician-directed or fixed-dose steroid replacement strategies for incomplete dexamethasone dosing prior to docetaxel chemotherapy (REaCT-DEX) [37] NCT02815319 [Internal funds]	60 (1)	Completed	While not meeting the predefined criteria of improving the time from randomization to starting docetaxel by 30 min, the fixed-dose replacement strategy reduced both the time to starting docetaxel and treatment variability. Fixed dosing of 8 mg of oral dexamethasone should be the preferred standard of care.
Adjuvant—Her-2 based therapies			
A randomized study comparing two standard of care chemotherapy regimens for lower-risk HER2-positive breast cancer (REaCT-Low Risk HER2) [38] NCT03705429 [London Regional Cancer Program Medical Oncology Research Fund (MORF)]	49 (2)	Completed	Feasibility endpoint was met. Rates of febrile neutropenia were higher (8.3% vs. 0%) in the docetaxel–cyclophosphamide plus trastuzumab vs. paclitaxel plus trastuzumab (APT) arm.
A randomized trial comparing 3- versus 4-monthly cardiac monitoring in patients receiving trastuzumab-based chemotherapy for early breast cancer (REaCT-EF) [39] NCT02696707 [Internal funds, donations]	200 (2)	Completed	Cardiac monitoring every 4 months was deemed non-inferior to that every 3 months.
Adjuvant—bisphosphonates			
A randomized trial comparing standard 6-monthly dosing of adjuvant zoledronate with a single one-time dose in patients with early-stage breast cancer (REaCT-ZOL) [40] NCT03664687 [CURE and the Ottawa Hospital Foundations]	211 (4)	Completed	Single infusion of zoledronate is associated with greater patient convenience and equivalent QoL, RFS, and OS outcomes.
Adjuvant—timing of radiation therapy and endocrine therapy trials			
A pragmatic randomized trial evaluating endocrine toxicity with concurrent versus sequential radiation and endocrine therapy in early-stage, hormone responsive breast cancer (REaCT-RETT) [41] NCT03948568 [Internal funds, donations]	262 (3)	Completed	No difference in endocrine therapy toxicity from baseline to 3 months and no difference in quality of life, compliance, or radiotherapy toxicity at twelve months.
Palliative Care			
A pilot randomized trial comparing 2 oral magnesium supplements for cancer treatment-induced hypomagnesemia (REaCT-Mg) [42] NCT02690012 [Patient Quality and Safety Committee PQ&I Project Grant from The Ottawa Hospital Department of Medicine with matched funding from the Division of Medical Oncology]	15 (1)	Completed	Despite oral magnesium tolerability and meeting most of its feasibility endpoints, this study did not meet its target accrual rate. Alternative designs are necessary for a definitive efficacy study.
A randomized trial of 4- versus 12-weekly administration of bone-targeted agents in patients with bone metastases from breast or castration-resistant prostate cancer (REaCT-BTA) [43,44] NCT02721433 [Internal funds, Canadian Institute of Health Research Grant, Cancer Care Ontario Grants, Donations]	263 (5)	Completed	The 12-weekly arm was non-inferior to the 4-weekly arm in terms of physical functioning. No statistically significant differences in secondary outcomes were observed, including pain, global health status, skeletal-related events, and toxicity.

**Table 2:** REaCT clinical trials portfolio—trials ongoing or closed to accrual.

Study Name Trial Number [Funding Source]	Number of Participants Enrolled (Number of Sites opened)	Current Status	Key Findings
Surgical—colorectal studies			
A pragmatic, randomized trial comparing no preparation versus preoperative oral antibiotics alone for surgical site infection rates in elective colon surgery (REaCT-NSQIP) [45] NCT03663504 [TOHAMO Innovation Grant]	439 (5)	Ongoing accrual	To come
Adjuvant—endocrine therapy and supportive care studies			
A pragmatic, randomized trial evaluating the risks and benefits of hormonal therapy in patients with low risk breast cancer who are 70 years of age and older (REaCT-70) NCT04921137 [TOHAMO Innovation Grant]	107 (7)	Accrual completed	To come
A pragmatic, randomized trial evaluating an endocrine therapy dose–frequency escalation strategy and its effects on tolerability and compliance (REaCT-TEMPO) NCT05754528 [Internal Funds, donations]	240 (2)	Accrual completed	To come
Adjuvant—chemotherapy supportive care studies			
A randomized pragmatic trial evaluating omission of granulocyte colony-stimulating factors in breast cancer patients receiving paclitaxel portion of dose-dense adriamycin–cyclophosphamide and paclitaxel chemotherapy (REaCT-OGF) NCT05753618 [TOHAMO Innovation Grant]	105 (3)	Ongoing accrual	To come
Neoadjuvant—Her-2-based therapies			
A prospective study evaluating 6 months of trastuzumab in patients with HER2 positive early-stage breast cancer (REaCT-HER TIME) NCT04928261 [The CURE Foundation]	26 (1)	Ongoing accrual	To come
Adjuvant—breast cancer well follow-up strategies			
A randomized trial evaluating personalized vs. guideline-based follow-up strategies for patients with early-stage breast cancer (REaCT-WELLNESS) NCT05365230 [TOHAMO Innovation Grant]	237 (1)	Accrual completed	To come
Supportive Care—bone metastases			
A pragmatic, randomized trial to evaluate the efficacy and safety of either continuing or further de-escalating bone modifying agents (BMA) after a minimum of two years of BMA in patients with bone metastases from breast cancer and castration-resistant prostate cancer (REaCT-HOLD) NCT04549207 [TOHAMO Innovation Grant]	240 (5)	Accrual completed	To come
Primary brain tumours			
A prospective observational study evaluating therapeutic outcomes related to gut microBIOME in glioblastoma (GBM) patients receiving chemo-radiation (Therabiome GBM) NCT05326334 [Gavin Murphy Fund]	12 (1)	Ongoing accrual	To come
